# Medical News

**Published:** 1853-05

**Authors:** 


### MEDICAL NEWS.

Delegates to the State Medical Society.—At a meeting of the Philadelphia County Medical Society, held April 20th, 1853, the following were elected delegates to the Pennsylvania State Medical Society: Wm. Ashmead, John B. Biddle, Henry Bond, Thomas F. Betton, J. R. Bryan, Chas. F. Bibighaus, B. H. Coates, Jos. Carson, D. Francis Condie, Wm. Darrach, G. Emerson, Jas. V. Emlin, Geo. Fox, P. B. Goddard, David Gilbert, Ed. Hallowell, S. L. Hollingsworth,

Isaac Hays, N. L. Hatfield, Samuel Jackson, W. Jewell, E. Janvier, A. L. Kennedy, W. H. Klapp, W. L. Knight, John P. Lamb, R. J. Levis, Wm. Mayburry, John K. Mitchell, Geo. W. Norris, A. Naudain, Hen. S. Patterson, L. Rodman, N. C. Reid, M. B. Smith, A. E. Stocker, L. Turnbull, F. West, J. Warrington, Thomas S. Yardley.

The Philadelphia Delegation to the National Medical Association.—The various bodies entitled to representation in the American Medical Association, have, so far as we have learned, selected the following delegates:

*College of Physicians.*—I. Hays, G. Fox, H. Bond, W. S. W. Ruschenberger, G. Emerson, J. R. Paul, C. Evans, W. Ashmead, J. Bell, E. Hallowell, A. Stillé, J. Carson, R. La Roche, E. Hartshorne, R. Bridges.

*Philadelphia County Medical Society.*—W. Ashmead, T. F. Betton, C. F. Bibighaus, D. F. Condie, B. H. Coates, J. Carson, G. Fox, S. Jackson, W. Jewell, J. F. Lamb, E. F. Leake, C. D. Meigs, W. Mayburry, G. W. Norris, H. S. Patterson, W. B. Page, L. Rodman, J. M. Thatcher, F. West, T. H. Yardley.

*Northern Medical Association.*—L. Curtis, B. S. Janney, N. L. Hatfield, J. F. Lamb, R. J. Levis, I. Remington

*University of Pennsylvania.*—S. Jackson, R. E. Rogers.

*Jefferson Medical College.*—J. K. Mitchell, J. Pancoast.

*Pennsylvania Medical College.*—J. M. Allen, J. J. Reese.

*Medical Institute of Philadelphia.*—J. B. Biddle.

*Philadelphia Medical Association.*—Wm. V. Keating.

*Pennsylvania Hospital.*—

*St. Joseph's Hospital.*—H. H. Smith.

*Hospital of P. E. Church.*—F. G. Smith.

Dr. Marshall Hall.—We have great pleasure in presenting to our readers, in the present number, the Lecture delivered by Dr. Marshall Hall before the Smithsonian Institute. Dr. Hall's visit to the United States will, we trust, afford frequent opportunities to the profession of becoming personally acquainted with one whose name and works have been so long and so favorably known to them.

University of Virginia.—Dr. Wm. B. Rogers has resigned the chair of Natural Philosophy, and Dr. J. Lawrence Smith that of Materia Medica and Chemistry in this Institution.

The Queen has conferred the honor of a baronetcy upon Dr. Henry Holland, one of the physicians-extraordinary to her Majesty.

Tribute to Dr. Brown-Séquard.—At a meeting of the members of the class attending Dr. Brown-Séquard's second course of lectures in Philadelphia, held at the Pennsylvania College, Ninth street, below Locust, on Tuesday, April 26th, 1853, the following preamble and resolutions were presented by Dr. Edward Hartshorne, and unanimously adopted:

*Whereas,* The second of the two courses of lectures delivered by Dr. Brown-Séquard in this city, has just closed; and,

*Whereas,* The members of the second class, most of whom were also members of the first, are unwilling to part with him, without expressing in some way their appreciation of his most interesting and instructive demonstrations, and of his many new views in biology, and the application of these to the practice of medicine and surgery, therefore—

*Resolved,* That we are under the greatest obligation to the learned and able lecturer, for his admirable series of experiments, so happily explained and illustrated, not only on account of the valuable instruction he has thus conveyed to us, but on account of the powerful impulse to the more efficient practical study of biological science, which his precept and example have thus afforded us.

*Resolved,* That we regard his visit to this country, and the delivery of the various brilliant and successful courses on his favorite subject, in the cities of Boston, New York and Philadelphia, as likely to mark a new era in the progress of biological research in the United States.

*Resolved,* That we take leave of Dr. Brown-Séquard, as our teacher, with great regret, not only on account of the many original views which he has presented to us, as the result of his own investigations, and the many useful applications of these views to the improvement of the practice of medicine and surgery, but on account of his attractive qualities as an amiable and honorable man. And finally,

*Resolved,* That in view of his contemplated return to his old home, and the field of his early triumphs, we cannot do better than to wish him the same distinguished success that attended him there, increased opportunity of further discovery, and a more lasting prosperity in all the relations of life.

On motion, the Chairman and Secretary were appointed a committee to convey a copy of the above Resolutions to Dr. Brown-Séquard, on behalf of the class, and to procure their publication in the Medical News and the Medical Examiner.

(Signed) Francis G. Smith, M.D., *Chairman.*

J. Cheston Morris, *Secretary.*

Glasgow Medical Journal.—The first number of a new and very able Quarterly Journal appeared on the 1st of April last, at Glasgow, in Scotland.

Cholera.—A recent letter states that there were 201 cases of cholera in St. Petersburgh, and that at the date of the letter there were thirty-two new cases, ten cures, and twelve deaths. This disease is disappearing at Breslau through (as the medical officers state) the low temperature of the atmosphere.—*Lond. Lancet, April 9th.*

Appointment.—M. Dubois has been appointed accoucheur to the Empress of the French.

Typhus Fever in Paris.—This frightful disease, according to the latest intelligence, is raging in Paris and its vicinity, and more especially in the military hospitals. Messrs. Begin and Levy have been ordered, as the medical inspectors, to make an official report of its origin and progress.

Academie de Medecine.—The Académie de Médecine yesterday elected as foreign members MM. Buffalini, of Florence, Mott, of New York, Warren, of Boston, Riberi, of Turin, Vieminckz, of Brussels, Retzius, of Stockholm, and Simpson, of Edinburgh.—*Galignani's Messenger*, 12*th March*, 1853.

Diseases Among the Bedouin Arabs.—Diseases are rare among them; and the epidemics, which rage in the cities, seldom reach their tents. The cholera, which has of late visited Mosul and Bagdad with fearful severity, has not yet struck the Bedouins; and they have frequently escaped the plague, when the settlements on the borders of the Desert have been nearly destroyed by it. The small-pox, however, occasionally makes great ravages among them, vaccination being still unknown to the Shammar; and intermittent fever prevails in the autumn, particularly when the tribes encamp near the marshes in Southern Mesopotamia. Rheumatism is not uncommon, and is treated, like most local complaints, with the actual cautery, a red-hot iron being applied to the part affected. Another cure for rheumatism consists in killing a sheep, and placing the patient in the hot reeking skin. Ophthalmia is common in the Desert, as well as in other parts of the East, and may be attributed as much to dirt and neglect as to any other cause.

The Bedouins are acquainted with but few medicines. The Desert yields some valuable simples, which are, however, rarely used. Dr.

Sandwith, hearing from Suttum that the Arabs had no opiates, asked what they did with one who could not sleep. "Do!" answered the Sheikh, "why, we make use of him, and set him to watch the camels." If a Bedouin be ill, or have received a wound, he sometimes comes to the nearest town, to consult the barbers, who are not unskilful surgeons. Hadjir, one of the great chiefs of the Shammar, having been struck with a musket ball, which lodged beneath the shoulder blade, visited the Pasha of Mosul to obtain the aid of the European surgeons attached to the Turkish troops. They declared an operation to be impossible, and refused to undertake it. The Sheikh applied to a barber who, in his shop in the open bazaar, cut quietly down to the ball, and taking it out, brought it to the Pasha in a plate, to claim a reward for his skill. It is true that the European surgeons in the service of the Porte are not very eminent in their profession. The Bedouins set broken limbs by means of rude splints.

The women suffer little in labor, which often takes place during a march, or when they are far from their encampment, watering the flocks or collecting fuel. They allow the children to remain at the breast until they are nearly two or even three years old, and, consequently, have rarely many offspring.—*Dublin Med. Press,* from *Discoveries in Nineveh by A. H. Layard, M. P., in Association Medical Journal.*

Death of M. Orfila—M. Orfila was but a short time ago snatched by the hand of death from the midst of friends and admirers, at the age of sixty-six years. This eminent man was so highly esteemed among his countrymen, and enjoyed such a world-wide reputation, that we gladly devote some of our space to sketch the brilliant career of so distinguished a member of our profession.

The deceased was born at Mahon, in the island of Minorca, on the 24th of April, 1787, and could, as well expressed by M. de Salvandy, from the rocks of his country contemplate both empires, and one day choose between the two lands. He possessed within himself the genius peculiar to each soil—the fertile, bold and searching activity of the one, the firmness, patience, and enduring perseverance of the other. Orfila's mind was first engaged upon the study of mathematics, as he was intended for a maritime life, at a time when the French and Spanish fleets were co-operating in the East. He actually served a short time, but soon relinquished the sea, and from the pursuit of mathematics turned to the cultivation of the natural sciences. For this purpose he repaired to the University of Valencia in the year 1804.

Physics and chemistry were at that period at a very low ebb in Spain; and Orfila, not satisfied with hearing his professors stating that air and water were two elements, seized upon the works of Lavoisier, Berthollet,

Fourcroy and others, and rose to the level of the discoveries of the day. But the University of Valencia was just then being threatened with abolition, on the plea of insufficient teaching; the professors provoked an inquiry, and put forward their pupils. Orfila greatly distinguished himself in the examinations, and so complete was his success, that the junta of Barcelona sent him to Paris at their own cost, to study chemistry, and the application of that science to the arts.

He arrived in Paris in 1807, and soon took a great liking to a country which, for the subsequent forty-six years, became his adopted land. The war which soon broke out between France and Spain deprived him of his allowance from Barcelona, but he was temporarily supported by a member of his family. It should here be mentioned that many years after this period, Orfila offered the junta his services, from gratitude at the patronage they had formerly granted him; but that body was in too disorganized a state to accept the honest man's disinterested proposals. Nor would his return to Spain have been a small sacrifice; for Vauquelin had welcomed him in his laboratory, and Fourcroy had entrusted to him the task of giving a few lectures on Organic Chemistry.

Orfila now obtained the degree of Doctor of Medicine, and began to lecture privately on Chemistry, Forensic Medicine, and even Anatomy, (a kind of authorized grinding,) and in this first and small laboratory he laid the foundation of the new science of toxicology. We say *new*, and advisedly; for the books published before Orfila's time mention none of those delicate tests introduced by the great chemist; it is true that even then certain poisons could be detected when dissolved in, or mixed with, water; but when these poisons were added to wine, milk, bile, &c., they were beyond the skill of the chemists of the day. Orfila's researches rendered investigations of this kind, and many others of equal importance, perfectly easy.

Orfila was soon appointed corresponding member of the Institute, (a generic name given to the four academies taken collectively,) and in 1819 he was appointed Professor of Forensic Medicine at the Faculty of Medicine, especially by the influence of the celebrated Hallé, who, though very ill, had himself carried to the medical school to vote for M. Orfila.

It was at first supposed that lectures on Forensic Medicine at the Faculty would attract but few hearers; but Orfila's happy mode of teaching, and of captivating the attention of the pupils, rendered the subject peculiarly attractive. His language was lucid, without being too much studied; and he treated the most difficult questions with so much method and precision, that the mind readily followed him. He was especially fond of conveying knowledge by actual experiment, and gave his explanation with a clear and penetrating tone of voice, in well chosen periods, and without too much fire or any hesitation. He loved to teach and guide the pupils, and thus drew around himself numerous and attentive hearers during the four years he lectured on forensic medicine, and the twenty-nine which he devoted to medical chemistry. This success is the more flattering, as pupils are not *bound* to attend the lectures of any professor, and that the latter have a fixed salary, independent of the number of students who may attend them.

The fame of Orfila was now daily spreading, and his works and teaching on toxicology, forensic medicine, and chemistry testify to his immense labors; but he seemed to direct his whole energy to the detection of poisons. He looked upon the science of toxicology as composed of two principal divisions—one comprising the symptoms of poisoning, the consequent lesion of texture, and the medical treatment; the other, more exclusively chemical, embracing the detection of the poison either with a view of the discovery of an antidote, or for guiding the verdict of a jury. Both these branches were so highly cultivated by Orfila, that he became the oracle in the courts of law, and the dread of those who contemplated the treacherous administration of deadly substances.

In 1820, the Academy of Medicine, as now constituted, was founded by Louis XVIII., and the original members were seventy in number. Orfila was one of them, and, strange to say, the youngest of all. He distinguished himself for the next thirty-three years as a fluent and able speaker, always defended the Academy in the contests which that learned body had to sustain, and, when chairman, acted with great tact and ability. Shortly before Orfila's death, only five of the original seventy members were left, and it was remarked that Orfila was again the youngest amongst them.

The revolution of 1830, which carried Louis Philippe to the throne, became, for Orfila, the beginning of a series of prosperous days, in which wealth, honors, and the most envied appointments were showered upon him. But it may be said to his praise, that he rendered the greatest services, both to the state and to learning, in the several responsible and eminent posts which he successively occupied.

As Dean of the Faculty of Medicine, (in which dignity he succeeded the celebrated Antoine Dubois, father of the present accoucheur, Paul Dubois,) he used his best efforts, for a period of eighteen years, to introduce improvements of great importance in the medical school. Without neglecting toxicology or his professional duties, he began to root up abuses, and to make regulations which to this day call for the gratitude of the students. Lectures, by his endeavors, were now given with regularity; the pupils were subjected to a wholesome discipline; the miserable building opposite the Faculty, was demolished, and a new one, called "Hôpital des Cliniques," erected in its place, where surgery and midwifery are carefully taught; new dissecting-rooms were substituted for the wretched places where anatomy was before cultivated; the Dupuytren museum was founded; a botanical garden opened; and the anatomical and pathological collections of the Faculty so enriched, that Orfila's name was attached to them as a lasting memorial of his wise and paternal government.

But Orfila was now called upon to take an active share in the higher branches of the administration connected with learning and medical science; his influence became immense, but he contrived to make many friends, and to render the greatest services by his great aptitude for public business, his vast experience, quick glance, and mastery of details.

Orfila successively took his seat in the Council General of Hospitals, and at the Board of Public Instruction. The profession gained much by his elevation, for it was through his exertions that the preparatory schools of medicine in various large provincial town were founded, and it was he who placed the examinations for the degree of Doctor of Medicine upon the present excellent footing.

The subject of this memoir obtained the usual marks of high favor at the hands of Government, and was successively made knight, officer, and commander of the Legion of Honor. He became accustomed to guide and regulate, and when the exalted man was thrust into private life by the destructive revolution of 1848, he felt the blow very acutely, and never recovered the shock.

Among all the claims which M. Orfila has to the gratitude of his countrymen there is one which will certainly be highly prized among us, and will remind our readers of those worthy members of our profession who, in England, have founded societies for the relief of the decayed medical man, his widow, or children. M. Orfila, at the period to which our narrative has arrived, founded a provident association for the benefit of medical men or their families; and for many years he was the President of the society. He constantly used his best efforts to promote its welfare, and succeeded in establishing it upon a sure and lasting basis. This society was not forgotten in the munificent legacies which M. Orfila made shortly before his death, and to which we shall presently allude.

But human prosperity is fragile; M. Orfila's career of usefulness and benevolence was arrested by the fall of Louis Philippe; he lost all his appointments, (except his professorship, which could not be taken from him,) and it is said that these changes have, to the hour of his dissolution, preyed upon his mind, and even hastened his death.

M. Orfila was not only displaced as Dean of the Faculty, but roughly called to account respecting the improvements and embellishments which had been made during his tenure of office; and the worthy and eminent man was, by envious detractors, sadly worried and taken to task. He endeavored to find comfort in scientific pursuits, and tried to regain his shattered health by a journey to the Pyrenees, but his friends saw that his powers were gradually failing.

Only a few weeks ago, M. Orfila resolved to make all his benevolent legacies before his death, a sort of defiance (as justly mentioned by Prof. Bérard) which the leveller of all human greatness resented by speedily snatching at his prey. More than £4000 were thus given by M. Orfila; firstly, to the Academy of Medicine, for prizes on medico-legal questions; secondly, to the Faculty of Medicine, for the preservation of the museum; thirdly, to the School of Pharmacy, for prizes; and to the Medical Benevolent Society for its praiseworthy purposes.

A very short time after these munificent gifts were announced, and whilst grateful addresses were being sent from all parts of France, M. Orfila was attacked with pneumonia, soon after his last lecture, delivered on the 4th of March. Drs. Chomel, Andral, and Rostan attended him with the greatest solicitude, but it was soon too plain that the illustrious patient must sink. He expired at half past seven in the morning, on the 12th of March, and had, thirty-six hours previously, sought the comforts of the Christian religion.

The funeral was as numerously attended as that of Dupuytren and Broussais; all the distinguished men in the arts, sciences, medicine, and the government, as well as crowds of students, accompanied the remains of this great and good man to their last and peaceful abode. The corners of the pall were held by M. Berard, Inspector of Public Instruction; M. P. Dubois, Dean of the Faculty of Medicine; M. Dubois, (d'Amiens,) Perpetual Secretary of the Academy of Medicine, and M. Bussy, Director of the School of Pharmacy. Orfila is buried close to the tombs of Lisfranc and Barruel, the latter of whom was his first and much revered teacher.

The works left by M. Orfila are the following: 1. On Forensic Medicine, with an Appendix on Legal Exhumations, with twenty-six plates, four vols., fourth edition in 1848; 2. Elements of Medical Chemistry, two vols., eighth edition in 1851; 3. On Toxicology, two vols., fifth edition in 1852; and a great number of papers and reports on Toxicology and Forensic Medicine, which have so largely contributed in placing both sciences on their present improved footing, and have gained for their author an imperishable fame.—*London Lancet.*

Death of Robert James Graves, M. D.—This distinguished Irish Physician, whose numerous and valuable contributions to Pathology and Practical Medicine, in the Medical Journals, have made his name familiar to the American as well as European profession, died in Dublin, on the 20th of March last, at the early age of 56.

He was the youngest son of a dignitary of the Irish church, the Rev. Richard Graves, D. D., Dean of Ardagh, and was educated at Trinity College, Dublin. He graduated in Medicine at the Dublin Medical School, and after passing three years in visiting the chief Continental Schools, in 1821, commenced the practice of medicine in Dublin. He soon took part in the establishment of a private school of medicine, and having been elected one of the Physicians of the Meath Hospital, entered with great energy and zeal on the arduous career of a medical teacher. The school of which he was one of the founders, known as the Park street School, (now the site of St. Mark's Ophthalmic Hospital,) rapidly acquired a very high character. Here he first taught medical jurisprudence, subsequently pathological anatomy, but afterwards became associated with Doctor, now Sir Henry, Marsh, in the Chair of Practice of Physic. The Meath Hospital, however, was the great theatre of his most important labors. Here he set himself vigorously to work to reform the existing system of medical education. Hitherto, the student had to depend on himself for the acquirement of a knowledge of disease. Books were written, and lectures delivered, both of which avail but little without that actual practical knowledge of the various phenomena of disease to be gained at the bedside alone. Some years previously, it is true, Dr. William Stokes had commenced the system of actually instructing the student by the bedside; but it remained for Dr. Graves thoroughly to incorporate clinical instruction with the other elements of medical education, and to cause its immense importance to be fully and generally understood and recognised. He soon found apt and zealous pupils, and many of the best and most accomplished practitioners now in Ireland, England, the Colonies, and the public service, were then numbered among Dr. Graves's class at the Meath Hospital, and many have lived to acknowledge with pleasure and pride the obligations they owed to his teachings, and the stimulus which his example lent to their exertions. Two among the number must be specially named—Dr. Richard Townsend and Dr. William Stokes, now Regius Professor of Physic in the University of Dublin. Of the latter it is not here the time or place to speak. To the former we may be allowed to pay a brief but well deserved tribute in passing. Hurried away by an untimely death, the works which he has left only serve to show how deeply the Irish school has to deplore the loss of one whose early labors gave such sure presage of a brilliant and successful career. Those acquainted with the literature of thoracic pathology will not need to be reminded of his essays in the "Cyclopædia of Medicine."

Thus energetically and ardently working and teaching, the example of Dr. Graves at this period exercised the best influence on the medical youth of Dublin. His labors, however, were not confined to those of teaching. From an early period in his medical career he evinced the highest talents for original observation, and the results of his inquiries began to appear in print, and to attract attention, from the masterly style of his delineations of disease, his graphic manner, and the clearness, judgment, and decision with which his views were enunciated.

Dr. William Stokes having graduated in Edinburgh, and having subsequently been appointed Dr. Graves's colleague at the Meath Hospital, these two names are henceforth to be met with together as teachers and fellow-laborers in the field of original research. Under their joint editorship appeared the valuable series of Meath Hospital Reports, which have connected the name of this institution with the progress of Irish medicine during the last thirty years.

In the year 1827, Dr. Graves was elected Professor of the Institutes of Medicine to the King and Queen's College of Physicians in Ireland,—a chair which he continued to fill for many years with great distinction. One of his most distinguished pupils thus speaks of Dr. Graves's success as a teacher in this department:—"I am glad to acknowledge my own obligations to the similar chair (Institutes of Medicine) in the University of Dublin, filled then by my distinguished friend, Professor Graves, well known throughout Europe by his contributions to physiology and clinical medicine. From him I first imbibed a taste for physiological inquiry; and under his guidance and direction my first studies upon that subject were pursued."—*Dr. Todd's Farewell Address.*

As a reformer in practice, Dr. Graves has done invaluable service; and in no respect more so than as regards the treatment of the typhus fever of Ireland. This disease, always endemic in the sister island, occasionally breaks forth as an epidemic visitation of the most fatal kind, and several years are popularly memorable as "the fever years." Such were 1817, 1822, and lastly 1846–47. Having enjoyed ample opportunities of studying this fatal pestilence, Dr. Graves became not less distinguished as a practitioner than as a teacher and propagator of bold and enlightened views in the treatment of fever; and on no occasions were his *cliniques* at the Meath Hospital better attended than when it was known that fever was to be his theme. The views of treatment which prevailed at the period when Dr. Graves entered on practice were decidedly in favor of the lowering and depleting plan, whether by purgation or otherwise. In this respect, practice differed then very much from what it had been in the latter end of the preceding century. In the days of Harvey, Purcell, Cleghorn, M'Bride, Plunkett, Egan, and Quin, the tonic plan was followed, and with success; wine and other stimulants were freely exhibited. The propagation of the Hunterian pathology had, however, in subsequent years, filled the minds of men with antiphlogistic theories, to the exclusion of all others, and in these views the Dublin practitioners, with some honorable exceptions be it remarked, very freely shared up to the date of Dr. Graves's teachings. An intimate study of the disease, and a careful observation of the alarming symptoms of early prostration so common in Irish typhus, convinced Dr. Graves of the error of the practice in vogue, which consisted chiefly in withholding nourishment and administering purgatives. Against this system he took up arms, and waged a successful war, not, it may be imagined, without violent opposition. His views, however, soon gained converts, and, aided by his colleague and other enlightened practitioners, the old plan gradually gave ground to the new. There was nothing in which Dr. Graves took more real pleasure and pride than in the changes in practice thus brought about. "Let them write it as my epitaph, that I fed fevers," said he, on one occasion, to his colleague.

The careful support of the system by nourishment from an early period of the disease, and the courageous use of stimulants when indicated by symptoms of depression, now form the leading therapeutic principles in the management of fever cases with all the well-educated practitioners of the Irish school; but unquestionably to Dr. Graves is due the merit of promulgating these views, or at least of reviving the practice, which had fallen into disrepute since the days of the Quins and the Plunketts. Did our limits permit, we could dwell at considerable length on other important principles of treatment advocated by Dr. Graves. We can only refer to his papers "On the Use of Tartar Emetic and Opium in the Delirium of Fever," "The Employment of Acetate of Lead," etc. etc., all which will be found in his collected treatises on clinical medicine.

Independently of the publication of his various detached papers and monographs, Dr. Graves lent valuable assistance to the establishment of a periodical medical literature in Ireland. In the year 1830, the fifth and last volume of the "Dublin Hospital Reports" was committed to his editorship by Dr. Cheyne. Two years subsequently, the *Dublin Journal of Medical and Chemical Science,* the predecessor of the *Dublin*

*Quarterly Journal of Medicine*, was projected and established by Sir Robert Kane, then a student of medicine, and a pupil at the Meath Hospital. After the appearance of a few Numbers, Drs. Graves and Stokes became associated in the editorship of the periodical, and Dr. Kane having been soon forced to resign his connexion with it by his increasing devotion to chemical inquiry, it continued in the same hands till 1842.

In the original and review department of this journal, Dr. Graves was a large and constant contributor. In the year 1843, appeared the first edition of his "Clinical Lectures on the Practice of Medicine." Of this work, which passed through a second edition with much careful revision, and the addition of much valuable matter under the hands of Dr. Neligan in the year 1848, it is quite unnecessary for us to speak. It is well known to every clinical school in Europe.

As a lecturer, Dr. Graves was distinguished by a force and clearness of language, and an earnestness of manner, which irresistibly commanded attention, while his fine person and noble features won the admiration of his hearers. His style as a writer was at once simple yet nervous, and full of graphic power; and his delineations of disease are among the most successful of modern medical compositions. He was a strenuous advocate of the doctrine of contagion, and vigorously opposed the views advanced with regard to the non-contagious nature of cholera during its last outbreak. He was a firm supporter of the dignity and honor of his profession, and on the only occasion on which he descended into the arena of medical politics, he fought boldly and fearlessly, though unsuccessfully, for the rights of his brother practitioners. During many years Dr. Graves enjoyed a large and lucrative practice, and was much *recherché* as a consultant. For some time past his health had been only indifferent, and of late he suffered from attacks of atonic gout. His last illness was attended with considerable suffering, the lungs being much congested. He had also violent paroxysmal attacks of cough, which the slightest exertion was sufficient to induce. Symptoms of a purpuric condition of the blood, accompanied by anasarca, were subsequently manifested. These sufferings were borne with Christian fortitude and complete resignation. Dr. Graves expired in the forenoon of the 20th of March, at the age of 56.—*Lon. Med. Times & Gaz.*

